# Sex-specific associations of body composition measures with cardiac function and structure after 8 years of follow-up

**DOI:** 10.1038/s41598-021-00541-x

**Published:** 2021-10-26

**Authors:** Sharon Remmelzwaal, Joline W. J. Beulens, Petra J. M. Elders, Coen D. A. Stehouwer, M. Louis Handoko, Yolande Appelman, Vanessa van Empel, Stephane R. B. Heymans, A. Johanne van Ballegooijen

**Affiliations:** 1 Department of Epidemiology and Data Science, Amsterdam UMC, VU University Medical Center, Vrije Universiteit Amsterdam, Amsterdam Cardiovascular Sciences, De Boelelaan 1089a, 1081 HV Amsterdam, The Netherlands; 2 Julius Center for Health Sciences and Primary Care, University Medical Center Utrecht, Utrecht, The Netherlands; 3 Department of General Practice and Elderly Care Medicine, Amsterdam Public Health Research Institute, Amsterdam University Medical Center, Location VUmc, Amsterdam, The Netherlands; 4 Department of Internal Medicine, Cardiovascular Research Institute Maastricht (CARIM), Maastricht University Medical Center+, Maastricht, The Netherlands; 5 Department of Cardiology, Amsterdam UMC, Vrije Universiteit Amsterdam, Amsterdam, The Netherlands; 6 Department of Cardiology, Maastricht University Medical Center, Maastricht, The Netherlands; 7 Department of Cardiology, Maastricht University, CARIM School for Cardiovascular Diseases, Universiteitssingel 50, 6229 ER Maastricht, The Netherlands; 8 Centre for Molecular and Vascular Biology, Department of Cardiovascular Sciences, KU Leuven, Herestraat 49, bus 911, 3000 Leuven, Belgium; 9 Department of Nephrology, Amsterdam UMC, Vrije Universiteit Amsterdam, Amsterdam Cardiovascular Sciences, Amsterdam, The Netherlands

**Keywords:** Heart failure, Inflammation

## Abstract

We investigated the prospective associations of body composition with cardiac structure and function and explored effect modification by sex and whether inflammation was a mediator in these associations. Total body (BF), trunk (TF) and leg fat (LF), and total lean mass (LM) were measured at baseline by a whole body DXA scan. Inflammatory biomarkers and echocardiographic measures were determined both at baseline and follow-up in the Hoorn Study (n = 321). We performed linear regression analyses with body composition measures as determinant and left ventricular ejection fraction (LVEF), left ventricular mass index (LVMI) or left atrial volume index (LAVI) at follow-up as outcome. Additionally, we performed mediation analysis using inflammation at follow-up as mediator. The study population was 67.7 ± 5.2 years and 50% were female. After adjustment, BF, TF and LF, and LM were associated with LVMI with regression coefficients of 2.9 (0.8; 5.1)g/m^2.7^, 2.3 (0.6; 4.0)g/m^2.7^, 2.0 (0.04; 4.0)g/m^2.7^ and − 2.9 (− 5.1; − 0.7)g/m^2.7^. Body composition measures were not associated with LVEF or LAVI. These associations were not modified by sex or mediated by inflammation. Body composition could play a role in the pathophysiology of LV hypertrophy. Future research should focus on sex differences in regional adiposity in relation with diastolic dysfunction.

## Introduction

Heart failure with preserved ejection fraction (HFpEF) is an emerging public health problem. It affects more women than men^1^. HFpEF is often accompanied by multiple comorbidities, such as type 2 diabetes (T2D), hypertension, chronic obstructive pulmonary disease and adiposity^2,3^. Body mass index (BMI), waist circumference and waist-to-hip ratio are the most common indicators of adiposity and higher BMI, waist circumference and waist-to-hip ratio are associated with increased risk of incident HFpEF^4,5^. A different measure of adiposity is visceral fat and higher visceral fat is also associated with increased risk of HFpEF^5^. In a Mendelian randomization study, participants with genetically predicted higher fat mass index (fat mass divided by height squared) had an increased risk of heart failure (HF)^6^. Moreover, higher adiposity is associated with risk factors of HFpEF, such as type 2 diabetes and cardiovascular diseases^7,8^. In the Helius study, a large multiethnic Dutch cohort, various body composition measures, such as BMI, waist-hip ratio and waist circumference, and fat percentage, were associated with T2D prevalence in both women and men separately^7^. Additionally, results from the MORGEN project, one of the two Dutch cohorts in the European Prospective Investigation into Cancer and Nutrition project, show that higher BMI (≥ 30 kg/m^2^) and waist circumference resulted in respectively threefold and twofold higher risk of fatal cardiovascular disease in comparison to normal BMI and waist circumference, after 10 years of follow-up^8^.

Cross-sectional studies have shown that higher BMI, waist circumference, body and visceral fat, and lean body mass are associated with worsening of various echocardiographic measures related to cardiac structure and function such as left ventricular mass and left atrial volume^9–14^. These echocardiographic measures are among other measures, used as diagnostic criteria to detect HFpEF^15^. As these indicators of adiposity differ between women and men^16^, difference in body composition might explain sex differences in development of HFpEF. However, only one study reported sex-stratified results for the associations between fat mass and echocardiographic measures, such as left ventricular mass, showing that a higher fat mass was associated with higher left ventricular volume in women, and with lower left ventricular volume in men^9^.

A proposed mechanism by which adiposity could lead to HFpEF development is via higher levels of systemic inflammation^17^. Though sex-specific results from cross-sectional studies on various body composition measures and levels of inflammatory biomarkers are inconsistent^18–20^.

However, body mass index (BMI) is an indirect measure of adiposity and introduces misclassification because it does not reflect changes in body fat that occur with age^21^. Additionally, BMI cannot differentiate between the proportion of fat and lean or muscle mass and is influenced by physical activity level^22^. Therefore, more specific measures for adiposity than BMI should be used, such as body fat in absolute or relative numbers. Most importantly, studies determining the prospective associations between body composition measures and echocardiographic measures related to cardiac structure and function are lacking. Moreover, it is unclear whether these associations differ for women and men and whether inflammation is a mediator in these associations.

Therefore, we aimed to determine the prospective association between body composition measures and echocardiographic measures related to cardiac structure and function. Second, we investigated whether sex was an effect modifier in these associations. Third, we determined the mediating effect of inflammation on the association between body composition measures and echocardiographic measures.

## Methods

### Study population

This study included participants with baseline and follow-up echocardiographic measurements of the Hoorn study^23^. The Hoorn Study is a prospective cohort, which started in 1989 with 2484 participants, and was initiated to study the prevalence and determinants of type 2 diabetes in the general population. In 1513 participants the baseline measurements were repeated during the first follow-up visit in 1996–1998. We included 831 participants who underwent echocardiographic measurements during the second follow-up visit in 1999–2001, considered as baseline for this study. This subgroup was oversampled for participants with impaired glucose metabolism (IGM) and T2D to enable investigation of effect-modification by glucose metabolism status^23^. Participants with missing information on dual-energy X-ray absorptiometry (DXA) parameters (N = 150), on low-grade inflammatory biomarkers (N = 27), on either left ventricular ejection fraction (LVEF), left ventricular mass index (LVMI) or left atrial volume index (LAVI) available (N = 16), or a combination of these three (N = 59) were excluded. After eight years of follow-up (fourth follow-up visit in 2007–2009), follow-up echocardiographic measurements were performed in 340 participants (239 participants were lost to follow-up, of whom 93 died, 39 had physical or mental health problems, 29 were untraceable or moved out of the area and 78 had other or unknown reasons). Participants with missing information at follow-up were excluded as well: low-grade inflammatory biomarkers at follow-up (N = 3) or without either LVEF, LVMI or LAVI available (N = 16), resulting in an analytic sample of 321 participants. We did not have complete data on all three echocardiographic outcomes, which resulted in different analytic samples for each outcome measure: 244, 250 and 268 participants for LVEF, LVMI and LAVI, respectively.

All individuals gave written informed consent, and the ethical committee of the VU University Medical Centre, Amsterdam, The Netherlands approved the study. All methods were performed in accordance with the relevant guidelines and regulations.

### Body composition

At baseline, a whole body DXA scan was performed using the fan beam technology (QDR-2000, software version 7.20D, Hologic, Brussels, Belgium). This equipment used an X-ray tube with a switched pulse stable dual energy radiation with two excitation voltages of 70 and 140 kV. The machine performs serial transverse scans from head to toe at 1.2 cm intervals providing a pixel size of 1.9 mm × 1.2 cm. For each pixel, this software calculates weight, bone mass and fat percentage. Lean tissue mass was indirectly calculated as weight minus bone mass and fat mass. All measurements and calculations were performed for the total body and specific regions: head, trunk, arms and legs. These regions were distinguished using anatomic landmarks as described previously^24^. All DXA scans were performed and read by one investigator. In the analyses we used a percentage of total body fat, trunk and leg fat mass of respectively total trunk or leg mass (trunk or leg fat mass/total trunk or leg mass × 100), and total lean mass as the determinants.

### Biomarkers

Fasting, venous blood samples were drawn at baseline and follow-up by trained nurses. Biomarkers of low-grade inflammation [C-reactive protein (CRP), serum amyloid A (SAA), interleukin 6 (IL-6), interleukin 8 (IL-8), tumour necrosis factor α (TNF-α) and soluble intercellular adhesion molecule 1(sICAM-1)], were measured by a multi-array detection system based on electro-chemiluminescence technology (MesoScaleDiscovery, SECTOR Imager 2400, Gaithersburg, Maryland, USA). All serum samples were measured in duplicate. Intra- and inter-assay coefficients of variability were for CRP, 2.8 and 4.0%; for SAA, 2.7 and 11.6%; for IL-6, 5.6 and 13.0%; for IL-8, 5.6 and 12.2%; for TNF-α, 3.9 and 8.8%; for sICAM-1, 2.4 and 4.9%; respectively.

### Echocardiographic measurements

Experienced ultrasound technicians unaware of the medical history of the participant performed the echocardiographic measurements at baseline and follow-up. These measurements were obtained according to a standardized protocol consisting of two-dimensional, M-mode and pulsed-wave Doppler assessment^25,26^. An experienced cardiologist evaluated the echocardiograms in order to ensure the quality. Left ventricular systolic dysfunction was determined by LVEF(%). Left ventricular mass indexed to height to the power of 2.7 (LVMI, g/m^2.7^) was determined to assess cardiac structure. For LV diastolic dysfunction left atrial volume index (LAVI) (mL/m^2^) was determined.

### Covariates

Study personnel collected demographic, metabolic and other risk factors, and medication use with standardized methods during the baseline and follow-up visits. Smoking status was categorized in never smokers, former smokers and current smokers. Educational level was self-reported and was stratified into three categories: low (no or primary education), middle (secondary education) and high (tertiary education). History of cardiovascular disease (CVD) was based on self-report or medical records. Blood pressure (mmHg) was measured twice at the left upper arm in a sitting position using an oscillometric device and averaged (Collin Press-Mate, BP-8800). All participants in the Hoorn Study, except those with previously diagnosed T2D, underwent a standard oral glucose tolerance test and were classified as either NGM, IGM (either impaired fasting glucose or impaired glucose metabolism), or T2DM according to the 1999 WHO criteria)^27^. Estimated glomerular filtration rate (eGFR) (mL/min/1.73 m^2^) was calculated according to CKD-EPI 2009 [Chronic Kidney Disease Epidemiology Collaboration] formula^28^.

### Statistical analyses

Baseline characteristics are presented as mean ± SD, median [interquartile range] in case of a skewed distribution, or number (percentages) for the total population and stratified by sex. All analyses were performed in Rstudio version 3.5.3.

#### Linear regression analyses

We performed linear regression analysis using body composition measures, in percentages, at baseline as determinant and either LVEF, LVMI or LAVI at follow-up as the outcome to determine the prospective association between body composition measures and echocardiographic measures at follow-up. These analyses were adjusted for the respective echocardiographic measures at baseline, to account for baseline differences, and follow-up duration (years). We reported unstandardized regression coefficients per ten percentage points increase of body composition measures, and their respective 95% confidence intervals for the total population and stratified by sex. An increase of ten percentage points reflects the standard deviation and is used for ease of interpretation of the regression coefficients.

#### Confounder selection

Potential confounders were selected a priori and included age, sex, smoking status, (residual) kidney function, hypertension status, CVD and anti-inflammatory medication use, all measured at baseline. Anti-inflammatory medication can influence certain inflammatory markers and include nonsteroidal anti-inflammatory drugs (NSAIDs), platelet aggregation inhibitors (abciximab), lipid lowering agents (statins and niacins) and ACE inhibitors^29^.

In the first model, age, sex and glucose metabolism status at baseline, were added as potential confounders. In the second model, we additionally added HbA1c, kidney function, hypertension status, history of CVD and smoking status, all measured at baseline. Additionally, sex was assessed as effect modifier by including interaction terms in all models (body composition measures × sex). ANOVA was used to determine improved model fit for the model with and without the interaction term. A P-value of < 0.05 indicated a better model fit.

#### Calculation inflammation standard scores

Individual biomarker levels were divided into a combined Z-score of low-grade inflammation, for both baseline and follow-up. When biomarker levels were not normally distributed, the levels were natural log-transformed. The Z-scores were calculated per individual as follows: (individual value − study population mean)/study population standard deviation. The individual Z-scores were averaged into an overall Z-score for baseline and follow-up. Higher scores indicate more inflammation. The Z-score represents the deviation from the mean per standard deviation: 0 no deviation, 1 represents 1 standard deviation (SD) larger than mean.

#### Mediation analysis

**Figure 1:**
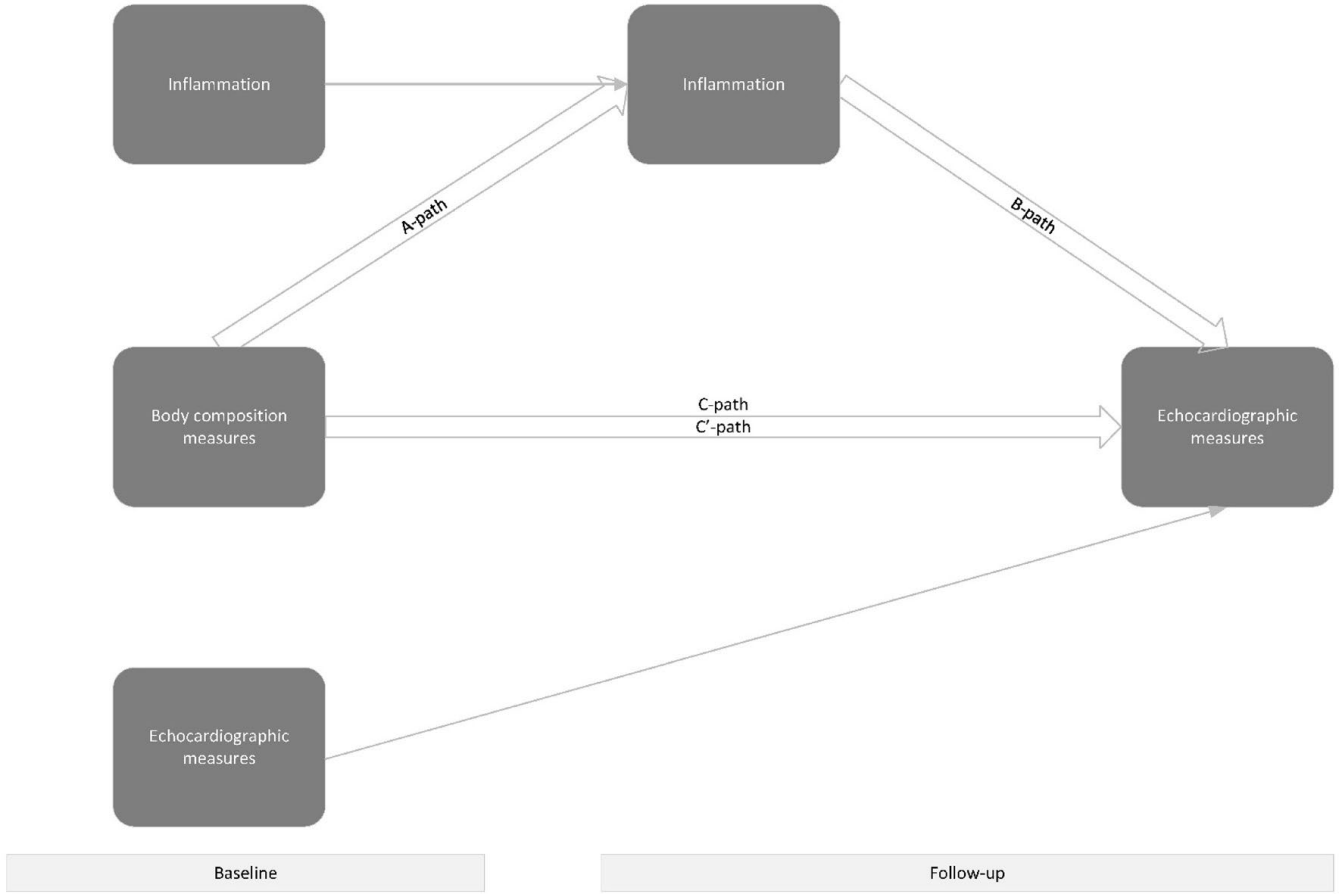
Directed Acyclic Graph of the relationship between trunk fat at baseline and echo parameters at follow-up, as mediated by inflammation at follow-up.

We performed mediation analysis using body composition measures, in percentages, as determinant, the continuous biomarker Z-score at follow-up, as mediator, and either LVEF, LVMI or LAVI at follow-up as the outcome, as visualized by a directed acyclic graph (Fig. 1). The following formulas were used for the calculation of the mediation paths to account for the longitudinal design and the repeated measurements of both mediators (continuous biomarker Z-scores) and outcomes (cardiac structure and function)^30^:

1 $$\text{A}-\text{path}: Biomarker \; Z-score \; at \; follow-up = body \; composition \; measures \; at \; baseline + biomarker \; Z-score \; at \; baseline + cardiac \; structure \& function \; at \; baseline + follow-up \; time,$$

2 $$\text{B}-\text{path} \; \& \; C{^{\prime}}\text{path}: Cardiac \; structure \& function \; at \; follow-up = body \; composition \; measures \; at \; baseline + cardiac \; structure \& function \; at \; baseline + biomarker \; Z-score \; at \; baseline + Z-biomarker \; at \; follow-up + follow-up \;time.$$

Formula (1) estimates the association between body composition measures, in steps of ten percentage points increase of trunk fat, and biomarker Z-scores (a-path). Formula (2) estimates the association between the biomarker Z-scores and cardiac structure and function (b-path), and the direct effect (c′-path) of body composition measures on cardiac structure and function. The indirect effect was calculated by multiplying the a-path with the b-path. The total effect (c-path) of body composition measures, in steps of ten percentage points increase of the respective body composition measure, on cardiac structure and function was estimated by multiplying the direct effect and the indirect effect. R-package ‘lavaan’ was used to determine the indirect and directs effects^31^. We reported unstandardized regression coefficients, and their respective 95% confidence intervals. Proportion mediated was calculated by: $\frac{Indirect effect}{Indirect effect + total effect}$ and only if indirect effect was greater than the total effect, and if both effects were in the same direction.

#### Sensitivity analyses

First, we investigated selection bias due to loss to follow-up by comparing baseline characteristics of participants and dropouts, and a sensitivity analysis was performed using inverse-probability weighting for the second model. The numerator was calculated directly from the data as the probability of being in the study. The denominator was computed using logistic regression with complete data (yes/no) as the outcome and variables associated to missing data as independent variables (Supplementary Table 1). Second, we also added total body fat as additional potential confounder for the linear regression analyses with trunk and leg fat as determinants. Third, we calculated the determinants trunk and leg fat as trunk or leg fat mass/total fat mass × 100.

## Results

### Study population

The study population consisted of 321 participants (Table 1). The mean age at baseline was 67.7 ± 5.2 years and 50% were female. More men than women were current smokers: 19.9% versus 9.4%, respectively.

**Table 1:** Baseline population characteristics, DXA parameters and biomarkers of inflammations for 321 female and male participants of the Hoorn Study.

	Total (N = 321)	Women (N = 160)	Men (N = 161)
Age, years	67.5 ± 5.1	67.4 ± 5.1	67.7 ± 5.2
BMI, kg/m^2^	27.0 ± 3.4	27.0 ± 3.7	26.9 ± 3.1
**Glucose metabolism status**			
Normal glucose metabolism	177 (55.1%)	93 (58.1%)	84 (52.2%)
Impaired glucose metabolism	91 (28.3%)	40 (25.0%)	51 (31.7%)
Type 2 diabetes	51 (15.9%)	26 (16.3%)	25 (15.5%)
Systolic blood pressure, mmHg	139 ± 20	139 ± 21	139 ± 18
Diastolic blood pressure, mmHg	83 ± 11	82 ± 12	83 ± 10
Hypertension	164 (51.1%)	83 (51.9%)	81 (50.3%)
Current smoker	47 (14.6%)	15 (9.4%)	32 (19.9%)
eGFR, mL/min/1.73 m^2^	81.6 ± 11.8	81.1 ± 11.6	82.1 ± 12.0
History of cardiovascular disease	150 (46.7%)	71 (44.4%)	79 (49.1%)
**DXA-scan**			
Total body fat, %	34 ± 9	41 ± 6	28 ± 6
Trunk fat, %	34 ± 9	39 ± 8	29 ± 8
Leg fat, %	34 ± 11	43 ± 7	25 ± 6
Total lean mass, %	63 ± 9	57 ± 6	69 ± 6
**Low-grade inflammation**			
CRP, mg/L	1.9 [0.9; 3.9]	1.8 [0.8; 3.5]	2.0 [1.2; 4.2]
SAA, mg/L	1.6 [1.0; 3.0]	1.9 [1.3; 3.1]	1.3 [0.8; 2.6]
IL-6, ng/L	1.4 [1.0; 2.1]	1.3 [1.0; 2.1]	1.4 [1.1; 2.0]
IL-8, ng/L	14.5 [11.4; 19.1]	14.5 [11.7; 19.2]	14.5 [11.3; 18.9]
sICAM-1, μg/L	247 ± 56	245 ± 49	249 ± 63
TNF-α, ng/L	8.2 [7.0; 9.8]	8.2 [7.0; 9.6]	8.1 [6.8; 9.9]
**Low-grade inflammation at follow-up**			
CRP, mg/L	1.7 [0.8; 3.9]	1.4 [0.8; 3.3]	2.0 [1.0; 4.6]
SAA, mg/L	1.8 [1.1; 3.1]	2.2 [1.5; 3.6]	1.5 [1.0; 2.7]
IL6, ng/L	1.6 [1.1; 2.4]	1.3 [1.0; 2.3]	1.7 [1.2; 2.5]
IL8, ng/L	10.2 [7.8; 12.5]	10.5 [8.0; 13.8]	9.7 [7.7; 11.7]
sICAM-1, μg/L	240 ± 55	238 ± 51	241 ± 58
TNF-α, ng/L	8.4 [7.1; 10.0]	8.1 [7.0; 10.0]	8.6 [7.2; 9.9]

Values are depicted as numbers (percentages); means ± standard deviations; medians [interquartile ranges].

*DXA* dual-energy X-ray absorptiometry, *BMI* body mass index, *eGFR* estimated glomerular filtration rate, *CRP* C-reactive protein, *SAA* serum amyloid A, *IL-6* interleukin-6, *IL-8* interleukin-8, *sICAM1* soluble intercellular adhesion molecule 1, *TNFa* tumor necrosis factor α.

Mean follow-up was 7.5 ± 0.5 years. Participants with follow-up measurements were younger (67.5 ± 5 years vs. 72.4 ± 7 years), less often had a history of CVD (47% vs. 59%), and T2DM (16% vs. 24%) than people without follow-up measurements (Supplementary Table 1).

#### Association between body composition and echocardiographic measures

At baseline, mean total body fat and trunk fat were 34 ± 9%, mean leg fat was 34 ± 11% and mean total lean mass was 63 ± 9%. Mean baseline LVEF was 63 ± 8%, mean baseline LVMI was 40 ± 12 g/m^2.7^ and mean baseline LAVI was 24 ± 8 mL/m^2^ (Table 1). In the total population, total body, trunk and leg fat were associated with LVMI with regression coefficients of 2.9 (0.8; 5.1)g/m^2.7^, 2.3 (0.6; 4.0)g/m^2.7^ and 2.0 (0.04; 4.0)g/m^2.7^ per ten percentage points increase in total body, trunk or leg fat, respectively (Table 2). In men, total body, trunk and leg fat were associated with LVMI with regression coefficients of 3.6 (0.7; 6.5) g/m^2.7^, 2.5 (0.5; 4.8) g/m^2.7^ and 3.5 (0.6; 6.5) g/m^2.7^ per ten percentage points increase, respectively. In women, total body, trunk and leg fat were not associated with LVMI with regression coefficients of 2.8 (− 0.4; 6.0) g/m^2.7^, 2.2 (− 0.4; 4.9) g/m^2.7^ and 1.8 (− 1.1; 4.6) g/m^2.7^ per ten percentage points increase, respectively. However, these differences between men and women were not statistically significant (P-values for improved model fit of 0.9, 0.7 and 0.4, respectively). In the total population, total lean mass was associated with LVMI with a regression coefficient of − 2.9 (− 5.1; − 0.7) g/m^2.7^ per ten percentage points increase in total lean mass. In men, total lean mass was associated with LVMI with a regression coefficient of − 3.7 (− 6.7; − 0.6) g/m^2.7^ per ten percentage points increase. In women, total lean mass was, albeit not statistically significant associated with LVMI with a regression coefficient of − 2.8 (− 6.1; 0.5) g/m^2.7^ per ten percentage points increase. This difference between men and women was not statistically significant (P-value for improved model fit of 0.97). All body composition measures were not associated with either LVEF or LAVI. We did not observe effect modification by sex for these associations (P-values for improved model fit > 0.3).

**Table 2:** Prospective associations of body composition measures with echocardiographic measures (LVEF, LVMI and LAVI).

	LVEF, %			LVMI, g/m^2.7^			LAVI, mL/m^2^		
	Total population (N = 244)	Women (N = 120)	Men (N = 124)	Total population (N = 250)	Women (N = 128)	Men (N = 122)	Total population (N = 268)	Women (N = 134)	Men (N = 134)
Baseline values	63 ± 8	64 ± 8	62 ± 7	40 ± 12	39 ± 10	41 ± 13	24 ± 8	24 ± 6	25 ± 9
Follow-up values	54 ± 10	55 ± 11	52 ± 9	42 ± 12	41 ± 13	42 ± 11	26 ± 12	25 ± 12	26 ± 12
**Total body fat (per 10 percentage points)**									
Model 1	0.2 (− 1.8; 2.2)	− 0.1 (− 3.1; 3.0)	0.4 (− 2.3; 3.0)	3.1 (1.0; 5.1)	2.7 (− 0.3; 5.8)	3.7 (0.9; 6.6)	1.5 (− 0.4; 3.4)	0.9 (− 2.0; 3.8)	2.2 (− 0.3; 4.7)
Model 2	0.1 (− 2.0; 2.2)	− 0.3 (− 3.5; 2.9)	0.3 (− 2.3; 3.0)	2.9 (0.8; 5.1)	2.8 (− 0.4; 6.0)	3.6 (0.7; 6.5)	1.3 (− 0.6; 3.2)	0.6 (− 2.4; 3.6)	2.0 (− 0.6; 4.6)
**Trunk fat (per 10 percentage points)**									
Model 1	0.2 (− 1.4; 1.7)	− 0.1 (− 2.5; 2.2)	0.4 (− 1.7; 2.4)	2.4 (0.7; 4.0)	2.1 (− 0.3; 4.6)	2.7 (0.5; 5.0)	1.0 (− 0.4; 2.5)	0.6 (− 1.7; 2.8)	1.4 (− 0.5; 3.3)
Model 2	0.1 (− 1.6; 1.7)	− 0.2 (− 2.7; 2.3)	0.3 (− 1.8; 2.3)	2.3 (0.6; 4.0)	2.2 (− 0.4; 4.9)	2.5 (0.2; 4.8)	0.9 (− 0.6; 2.4)	0.4 (− 1.9; 2.8)	1.2 (− 0.9; 3.2)
**Leg fat (per 10 percentage points)**									
Model 1	− 0.1 (− 2.0; 1.8)	− 0.4 (− 3.2; 2.3)	0.3 (− 2.5; 3.1)	2.2 (0.3; 4.2)	1.8 (− 0.8; 4.5)	3.4 (0.5; 6.3)	1.0 (− 0.8; 2.8)	0.5 (− 2.0; 3.1)	2.4 (− 0.2; 5.0)
Model 2	− 0.2 (− 2.1; 1.8)	− 0.9 (− 3.7; 2.0)	0.4 (− 2.4; 3.2)	2.0 (0.04; 4.0)	1.8 (− 1.1; 4.6)	3.5 (0.6; 6.5)	0.9 (− 1.0; 2.7)	0.2 (− 2.5; 2.8)	2.4 (− 0.3; 5.1)
**Total lean mass (per 10 percentage points)**									
Model 1	− 0.2 (− 2.3; 1.8)	0.1 (− 3.0; 3.2)	− 0.4 (− 3.1; 2.3)	− 3.09 (− 5.2; − 1.0)	− 2.7 (− 5.8; 0.4)	− 3.9 (− 6.8; − 0.9)	− 1.4 (− 3.4; 0.5)	− 1.0 (− 3.9; 2.0)	− 2.1 (− 4.7; 0.4)
Model 2	− 0.1 (− 2.3; 2.0)	0.3 (− 3.0; 3.5)	− 0.4 (− 3.1; 2.4)	− 2.9 (− 5.1; − 0.7)	− 2.8 (− 6.1; 0.5)	− 3.7 (− 6.7; − 0.6)	− 1.2 (− 3.3; 0.8)	− 0.6 (− 3.7; 2.4)	− 1.9 (− 4.6; 0.8)

Unstandardized regression coefficients (95%CIs) are reported per ten percentage points increase of total body, trunk and leg fat, and total lean mass.

Model 1 is adjusted for age, sex (for total population), glucose metabolism status at baseline and follow-up time. Model 2 is additionally adjusted for HbA1c, kidney function, hypertension status, history of CVD and smoking status, all measured at baseline.

*LVEF* left ventricular ejection fraction, *LVMI* left ventricular mass index, *LAVI* left atrial volume index.

#### Mediation analyses

Table 3 shows the associations of total body or trunk fat, at baseline, and cardiac structure and function, at follow-up, as mediated by low-grade inflammation, at follow-up. We did not observe consistent associations of total body or trunk fat with low-grade inflammation (a-path). Although we did find some significant associations in the b-, c- and c′-paths in model 2, no evidence was found for a mediating role of low-grade inflammation. We found no effect modification by sex for any of the paths (P-values for improved model fit > 0.1).

**Table 3:** Prospective associations of body composition with cardiac structure and function and mediation by low-grade inflammation and endothelial dysfunction in the Hoorn Study.

Outcome	Determinant	Effect of body composition on mediator (a-path) Β (95%CI)	Effect of mediator on outcome (b-path) Β (95%CI)	Total effect (c-path) Β (95%CI)	Direct effect (c′-path) Β (95%CI)	Indirect effect (a-path x b-path) Β (95%CI)	Proportion mediated effect %
LVEF, %	Total body fat (per 10 percentage points)	0.1 (0.01; 0.2)	− 0.2 (− 2.5; 2.1)	0.1 (− 1.9; 2.1)	0.1 (− 1.9; 2.1)	− 0.03 (− 0.3; 0.2)	N/A
	Trunk fat (per 10 percentage points)	0.06 (− 0.03; 0.1)	− 0.2 (− 2.5; 2.1)	0.04 (− 1.5; 1.6)	0.05 (− 1.5; 1.6)	− 0.01 (− 0.2; 0.1)	N/A
	Leg fat (per 10 percentage points)	0.1 (0.03; 0.2)	− 0.2 (− 2.5; 2.1)	− 0.2 (− 2.1; 1.7)	− 0.2 (− 2.1; 1.7)	− 0.03 (− 0.3; 0.3)	12.4%
	Total lean mass (per 10 percentage points)	− 0.1 (− 0.2; − 0.01)	− 0.2 (− 2.5; 2.1)	− 0.1 (− 2.2; 2.0)	− 0.1 (− 2.2; 2.0)	0.03 (− 0.3; 0.3)	N/A
LVMI, g/m^2.7^	Total body fat (per 10 percentage points)	0.1 (− 0.01; 0.2)	− 0.5 (− 2.7; 1.9)	2.9 (0.8; 5.0)	3.0 (0.9; 5.1)	− 0.1 (− 0.3; 0.2)	N/A
	Trunk fat (per 10 percentage points)	0.07 (− 0.02; 0.2)	− 0.3 (− 2.6; 2.0)	2.2 (0.6; 3.9)	2.3 (0.6; 3.9)	− 0.02 (− 0.2; 0.1)	N/A
	Leg fat (per 10 percentage points)	0.1 (0.03; 0.2)	− 0.4 (− 2.7; 1.9)	2.0 (0.05; 4.0)	2.1 (0.1; 4.0)	− 0.1 (− 0.4; 2.6)	N/A
	Total lean mass (per 10 percentage points)	− 0.1 (− 0.2; − 0.01)	− 0.4 (− 2.8; 1.9)	− 2.9 (− 5.1; − 0.8)	− 3.0 (− 5.2; 0.8)	0.1 (− 0.2; 0.3)	N/A
LAVI, mL/m^2^	Total body fat (per 10 percentage points)	0.1 (− 0.04; 0.3)	− 2.4 (− 4.6; − 0.3)	1.3 (− 0.6; 3.2)	1.6 (− 0.3; 3.5)	− 0.3 (− 0.7; 0.1)	N/A
	Trunk fat (per 10 percentage points)	0.1 (0.002; 0.2)	− 2.3 (− 4.5; − 0.2)	0.9 (− 0.6; 2.3)	1.1 (− 0.4; 2.5)	− 0.2 (− 0.5; 0.1)	N/A
	Leg fat (per 10 percentage points)	0.1 (0.04; 0.2)	− 2.4 (− 4.5; − 0.2)	0.8 (− 1.0; 2.6)	1.1 (− 0.7; 3.0)	− 0.3 (− 0.7; 0.1)	N/A
	Total lean mass (per 10 percentage points)	− 0.1 (− 0.3; − 0.04)	− 2.4 (− 4.6; − 0.3)	− 1.2 (− 3.2; 0.8)	− 1.5 (− 3.5; 0.4)	0.3 (− 0.1; 0.8)	N/A

a-path: association between body composition, in steps of ten percentage points increase of trunk fat, and mediating variable at follow-up adjusted for mediator at baseline, b-path: association between mediating variable at follow-up and cardiac structure and function at follow-up adjusted for mediator and cardiac structure and function at baseline, c-path: association between body composition, in steps of ten percentage points increase of trunk fat, and cardiac structure and function at follow-up adjusted for cardiac structure and function at baseline, c′-path: association between body composition, in steps of ten percentage points increase of trunk fat, and cardiac structure and function at follow-up adjusted for mediating variable and cardiac structure and function at follow-up, indirect effect: indirect effect of body composition on cardiac structure and function at follow-up through mediating variable at follow-up. Model is adjusted for age, sex, BMI, glucose metabolism status, HbA1c, kidney function, hypertension status, history of CVD and smoking status all measured at baseline. Proportion mediated is calculated if total effect is greater than indirect effect, and if both effects are in the same direction.

*LVEF* left ventricular ejection fraction, *LVMI* left ventricle mass index, *LAVI* left atrial volume index, *BMI* body mass index, *eGFR* estimated glomerular filtration rate, *CVD* cardiovascular disease, *SD* standard deviation.

#### Sensitivity analyses

Additional adjustment for total body fat in the associations of trunk and leg fat with LVMI altered the regression coefficients: 0.7 (− 3.9; 5.3) g/m^2.7^ and − 0.6 (− 4.0; 2.8) g/m^2.7^, respectively. Use of trunk and leg fat relative to total body fat as determinant, resulted in similar results as the additional adjustment for total body fat with regression coefficients of 0.8 (− 1.2; 2.7) g/m^2.7^ and − 1.1 (− 3.5; 1.2) g/m^2.7^, respectively.

Inverse-probability weighting resulted in slightly stronger associations than the main analysis (Supplementary Table 2).

## Discussion

In this prospective cohort, higher total body, trunk and leg fat at baseline were associated with higher LVMI after eight years of follow-up, and higher total lean mass was associated with lower LVMI at follow-up. No associations were found with LVEF or LAVI. These associations were not mediated by low-grade inflammation. Effect modification by sex was not apparent in any of the associations.

Our findings are in line with earlier studies showing that higher total body, trunk and leg fat, and total lean mass are associated with echocardiographic measures of cardiac structure^9,11–14^. However earlier studies used a cross-sectional design and other measures for adiposity and cardiac structure and function. One study showed that visceral fat was associated with measures of strain, which is a measure of myocardial function^11^. Another study showed that higher visceral or abdominal subcutaneous fat was associated with left ventricular end-diastolic volume, concentricity and left ventricular wall thickness, but not with left ventricular mass^13^. Two other studies also observed that higher body fat was associated with higher left ventricular mass and left ventricular end-diastolic volume, but used either cardiac magnetic resonance^9^ or bio-impedance measurements^12^ to determine fat mass. Our study shows that both total body, trunk and leg fat are prospectively associated with increased LVMI, but not LAVI and LVEF. We also showed that total lean mass is prospectively associated with decreased LVMI. Altogether, these studies consistently show an association of measures of body fat with increased left ventricular mass. The association with measures of diastolic and systolic function is limited and inconsistent, and requires further investigation.

One study reported sex-stratified results for the associations between fat and lean mass and echocardiographic measures, such as left ventricular mass and left ventricular volume^9^. Similar to our study, they observed that higher fat mass was associated with high left ventricular mass, in both men and women. However, the association between fat mass and left ventricular volume differed between women and men, showing that a higher fat mass was associated with higher left ventricular volume in women, and with lower left ventricular volume in men^9^. Furthermore, whereas we have found that higher lean mass was associated with lower LVMI, they observed that higher lean mass was associated with higher left ventricular mass. In our study, we observed a slightly stronger association in men, in comparison to women, for the associations of total body and trunk fat with LAVI, i.e. worsening of diastolic function. Altogether, there seems to be limited evidence that female predominance of HFpEF might be due to differences in fat and lean mass between men and women. However, sex differences in regional adiposity (epicardial and visceral fat versus subcutaneous fat), instead of aspecific fat mass, could be crucial to differentiate between the development of HFpEF in men and women.

Cellular mechanisms that drive the development of HFpEF, in general populations specifically, are unclear. One paradigm states that comorbidities, such as adiposity, induce pro-inflammatory cytokines that result in myocardial remodeling in the development of HFpEF^17^. Although high total body and trunk fat were associated with higher LVMI, confirming the potential role of adiposity in the development of LV hypertrophy and possibly HFpEF, we could not confirm the mediating role of low-grade inflammatory biomarkers in this association. This is in contrast to a cross-sectional study in the Hoorn Study that showed that higher total body and trunk fat were associated with higher low-grade inflammation levels^32^. However, since low-grade inflammation was not consistently associated with cardiac function, this did not explain the association of the body composition measures with LVMI. Specific fat depots, such as epicardial fat, i.e. regional fat that surrounds the myocardium, has pro-inflammatory effects and is higher in older women in comparison to men and younger women and could be one of the sex-specific mechanisms in the development of HFpEF^33,34^. Future research should focus on sex differences in regional adiposity in general populations and (a)symptomatic patients, in relation with diastolic dysfunction as a precursor of HFpEF, to further unravel the pathophysiological mechanisms and the course in the development of HFpEF. Furthermore, the role of regional adiposity in screening programs among general populations for the detection of early stages of HFpEF should be studied.

Our study is the first prospective study that determined the association of body composition measures at baseline with measures of cardiac structure and function at follow-up. Additionally, to our knowledge, this is the first time that the potential mediating effect of low-grade inflammation on this association has been studied. Several strengths are the long follow-up time of approximately eight years and the standardized measurements of biomarkers at two time points. Further, we presented both stratified results by sex and mediation analyses to provide more insight in the results. Nonetheless, there are certain limitations we need to address. Due to the follow-up time, we had a high loss to follow-up that could result in survival bias. However, a sensitivity analysis using inverse-probability weighting to adjust for this selection bias gave slightly stronger results as the main analysis meaning that healthier participants were included in this study and the main analysis would be an underestimation of the actual association. Second, we combined the inflammatory biomarkers in an overall low-grade inflammation Z-score. The underlying assumption is that all separate biomarkers reflect a similar pathophysiological mechanism. However, there is no consistent evidence whether this is true in a general population. Third, the DXA measurements were performed at baseline only, so changes over time could not be determined, and we did not have data available for visceral or subcutaneous fat. Fourth, other important echocardiographic measures for diastolic dysfunction, such as strain, were not measured in this cohort.

In conclusion, higher total body, trunk and leg fat at baseline was associated with higher LVMI, but not with LVEF and LAVI at follow-up. Higher total lean mass was associated with lower LVMI at follow-up. This could implicate the role of total body and trunk fat in the pathophysiology of LV hypertrophy. Low-grade inflammation is not a mediator in these associations. Effect modification by sex was not apparent in all associations. Future research should focus on sex differences in regional adiposity, in relation with diastolic dysfunction and HFpEF.

## Supplementary Information

Supplementary Tables.
