# Supplementary material for: Sex-specific associations of body composition measures with cardiac function and structure after 8 years of follow-up
Source: Sci Rep. 2021 Oct 26;11:21046. doi: 10.1038/s41598-021-00541-x (PMC8548503; doi:10.1038/s41598-021-00541-x)
Supplement: Supplementary file 1 — Supplementary Tables. [file 41598_2021_541_MOESM1_ESM.docx]

Supplementary table 1. Baseline characteristics of complete cases (N=321) versus loss to follow-up (N=258) of Hoorn Study participants.

|  | Hoorn Study | | |
| --- | --- | --- | --- |
|  | Complete cases (N=321) | Loss to follow-up (N=258) | P-value |
| Age, years | 67.5±5.1 | 72.4±7.0 | < 0.001 |
| Female | 160 (49.8%) | 130 (50.%) | 0.96 |
| BMI, kg/m^2^ | 27.0±4.0 | 27.1±4.0 | 0.65 |
| Glucose metabolism status  Normal glucose metabolism  Impaired glucose metabolism  Type 2 Diabetes | 177 (55.1%)  91 (28.3%)  51 (15.9%) | 107 (41.5%)  86 (33.3%)  62 (24.0%) | < 0.01 |
| Systolic blood pressure, mmHg | 139±20 | 145±21 | < 0.001 |
| Diastolic blood pressure, mmHg | 83±11 | 83±11 | 0.85 |
| Hypertension | 164 (51.1%) | 159 (61.6%) | 0.01 |
| Current smoker | 47 (14.6%) | 56 (21.7%) | 0.08 |
| eGFR, mL/min/1.73m^2^ | 81.6±11.8 | 78.0 ±14.4 | 0.001 |
| History of cardiovascular disease | 150 (46.7%) | 153 (59.3%) | <0.01 |
| DXA-scan | | | |
| Total body fat, % | 34±9 | 35±10 | 0.1 |
| Trunk fat, % | 34±9 | 35±10 | 0.2 |
| Low-grade inflammation | | | |
| CRP, mg/L | 1.9 [0.9;3.9] | 2.6 [1.4;5.0] | < 0.001 |
| Serum amyloid A, mg/L | 1.6 [1.0;3.0] | 1.8 [1.1;3.5] | 0.05 |
| IL-6, ng/L | 1.4 [1.0;2.1] | 1.7 [1.2;2.5] | 0.001 |
| IL-8, ng/L | 14.5 [11.4;19.1] | 15.5 [12.3;20.1] | 0.06 |
| sICAM-1, μg/L | 247±56 | 268±61 | < 0.001 |
| TNF-α, ng/L | 8.2 [7.0;9.8] | 8.5 [7.1;10.2] | 0.27 |
| Echocardiographic measures | | | |
| LVEF, % | 63.0±7.5 | 62.0±8.8 | 0.16 |
| LVMI, g/m^2.7^ | 40.2±11.7 | 44.5±14.3 | < 0.001 |
| LAVI, mL/m^2^ | 24.3±8.0 | 27.0±12.4 | < 0.01 |

Values represent percentages, means ± standard deviations and medians [IQR].

Analysis methods: Pearson Chi-Square, independent sampl.es T-test and Mann–Whitney U test

Abbreviations: BMI = body mass index; DXA = dual-energy X-ray absorptiometry; LVEF = left ventricular ejection fraction; LVMI = left ventricular mass index; LAVI = left atrial volume index.

Supplementary table 2. Sensitivity analysis of the prospective associations of body composition measures with echocardiographic measures using inverse probability weighting.

|  |  | LVEF, % | LVMI, g/m^2.7^ | LAVI, mL/m^2^ |
| --- | --- | --- | --- | --- |
| Total body fat (per 10 percentage points) | Inverse probability weighting | 0.2 (-1.9;2.4) | 3.7 (1.5;5.8) | 1.1 (-1.0;3.1) |
| Trunk fat (per 10 percentage points) | Additionally adjusted for total body fat | -0.2 (-4.6;4.2) | 0.7 (-3.9;5.3) | -0.5 (-4.8;3.8) |
|  | Inverse probability weighting | 0.1 (-1.6;1.7) | 2.8 (1.1;4.5) | 0.7 (-0.9;2.3) |
|  | Trunk fat relative to total body fat as determinant | -0.1 (-2.0;1.7) | 0.8 (-1.2;2.7) | 0.6 (-1.1;2.4) |
| Leg fat (per 10 percentage points) | Additionally adjusted for total body fat | -0.7 (-3.9;2.6) | -0.6 (-4.0;2.8) | -0.4 (-3.5;2.7) |
|  | Inverse probability weighting | 0.1 (-1.9;2.1) | 2.7 (0.7;4.7) | 0.6 (-1.3;2.6) |
|  | Leg fat relative to total body fat as determinant | -0.1 (-2.3;2.1) | -1.1 (-3.5;1.2) | -0.8 (-3.0;1.3) |
| Total lean mass (per 10 percentage points) | Inverse probability weighting | -0.2 (-2.4;2.0) | -3.7 (-5.9;-1.5) | -1.0 (-3.1;1.1) |

Unstandardized regression coefficients (95%CIs) per ten percentage points increase in total body, trunk and leg fat, and total lean mass are reported. Model is adjusted for age, sex, glucose metabolism status, HbA1c, kidney function, hypertension status, history of CVD, smoking status and follow-up time.

Abbreviations: LVEF = left ventricular ejection fraction; LVMI = left ventricular mass index; LAVI = left atrial volume index.
